# Longitudinal study of metacognition’s role in self-efficacy and hope development

**DOI:** 10.1038/s41598-024-80180-0

**Published:** 2024-11-26

**Authors:** Paweł Kleka, Hanna Brycz, Mariusz Zięba, Agnieszka Fanslau

**Affiliations:** 1 Department of Psychology and Cognitive Science, Adam Mickiewicz University, Poznań, Poland; 2 Department of Psychology, University of Gdansk, Gdańsk, Poland; 3 SWPS University, Warsaw, Poland

**Keywords:** Psychology, Human behaviour

## Abstract

Self-regulation is a critical component of adaptive functioning, and individual differences in traits like metacognitive self-awareness (MCS), general self-efficacy (GSE), and hope play significant roles in influencing this ability. Self-awareness of cognitive biases is particularly relevant as it may enhance one’s capacity to manage challenges and pursue goals effectively. Prior research suggests that higher levels of MCS are associated with improved self-regulation, greater self-efficacy, and stronger hope for success. This longitudinal study sought to explore the development of self-regulation across 3 years of college education, with a specific focus on the role of MCS in shaping the trajectories of GSE and hope. Over 400 undergraduates were assessed five times throughout this period. Growth curve and causal mediation analyses were employed to examine the extent to which MCS contributed to changes in GSE and hope. Our findings indicate that self-awareness of cognitive biases (MCS) significantly influences the development of self-efficacy. Participants with higher levels of MCS exhibited a slightly faster increase in coping skills than those with lower levels. Additionally, MCS moderated the development of hope: individuals with above-median MCS scores showed a faster increase in hope, whereas those with below-median scores experienced an irregular decrease. In conclusion, MCS serves as a valuable resource for developing self-efficacy and hope. However, the influence of real-life challenges on hope appears to depend on an individual’s metacognitive skills, with better outcomes observed among those with higher MCS.

## Introduction

Besides imparting knowledge, one of the long-term objectives of higher education is to cultivate critical thinking skills and shape students’ capacity for reflective and proactive approaches to social challenges. A crucial factor in achieving this aim is the development of metacognition, that is, an awareness of one’s own thought processes, which plays a crucial role in self-regulation and learning^1^–^4^. It is particularly intriguing to explore whether metacognition (i.e., metacognitive awareness of one’s own cognitive biases) can support psychological constructs such as general self-efficacy (GSE) and hope.

Self-efficacy refers to an individual’s belief in their ability to control actions, motivation, and thoughts, as well as to effectively pursue intended goals^1^. GSE assists individuals in assessing their abilities and chances of success, influencing their commitment and perseverance. On the other hand, hope is a motivational state related to the belief in one’s ability to persevere in the face of obstacles when pursuing goals^5^. Both constructs, GSE and hope, are crucial in the context of academic achievement.

The aim of this study was to investigate whether a high level of metacognition might serve as a predictor of increased self-efficacy and hope over time. Analysing the role of metacognition as a predictor of these constructs holds theoretical significance in the context of self-regulated learning theory, which posits that conscious control over one’s thought processes enhances the ability to effectively manage emotions and actions^6^, ^7^. We therefore hypothesised that higher levels of metacognition would be positively related to self-efficacy and hope over time.

### Perceived self-efficacy and its role in self-regulation

Perceived self-efficacy refers to an individual’s belief in their ability to influence the outcomes of their actions, which constitutes a central element of self-regulation and motivation in goal pursuit^1^. Bandura^6^ points out that self-efficacy affects the effort invested in action, persistence in the face of difficulties, and effectiveness in dealing with obstacles. Moreover, the level of self-efficacy conditions the emotions related to a particular activity and the stress threshold that an individual can endure^8^.

Experimental studies demonstrate a link between self-efficacy and task performance, with higher GSE contributing to better performance and greater satisfaction^9^. A high level of self-efficacy among school-aged youth contributes to academic success, improved well-being, and reduced stress levels^10^. Importantly, metacognition’s role partially mediates GSE’s effect on academic outcomes^11^. Skills associated with GSE, such as planning, evaluation, and focus, reinforce self-regulated learning processes, which are particularly crucial during adaptation to the academic environment^12^.

Contemporary research on GSE focuses not only on specific situations but also on general self-efficacy, that is, an individual’s belief in their ability to achieve goals regardless of context^13^–^15^. General self-efficacy (GSE) is a significant source of motivation in the process of independent learning and positively impacts students’ adaptation during their first academic year^16^.

### Hope as a motivational state in goal pursuit

Hope, according to Snyder and colleagues’ theory^17^–^19^, is a motivational state based on two interconnected beliefs: agency and pathways. Agency refers to the belief in one’s ability to initiate and sustain actions, while pathways represent persistence in finding ways to achieve goals. Hope, defined in this way, is particularly important in the face of difficulties—agency provides belief in sufficient resources to overcome obstacles, while pathways indicate the existence of ways out of situations.

Studies show a positive impact of hope on psychological adaptation^20^, positive affect^19^, and lower levels of depression^17^, ^21^, ^22^. Higher levels of hope are associated with better social relationships^17^, ^20^, better physical^23^–^25^ and mental health^26^, and greater life satisfaction^27^. Moreover, hope is positively correlated with academic performance at various levels of education^17^, ^19^, ^21^, ^28^, ^29^. Students and pupils with high levels of hope are more likely to continue their studies and complete their degree programmes than those with low hope, as confirmed by longitudinal studies^19^ and meta-analysis^30^.

Although hope and general self-efficacy (GSE) are correlated and both reflect positive expectations, Snyder considers them as distinct constructs, mainly due to the role of emotions^19^. Bandura’s theory of self-efficacy focuses on cognitive processes and expectations about the consequences of actions^6^, while the theory of hope encompasses the interaction of cognitive and emotional processes in goal pursuit^17^, ^31^, ^32^. Emotions play a crucial role in goal achievement, helping to form intentions and increasing motivation^19^.

Empirical studies show that agency, as a component of hope, is an independent predictor of well-being, distinct from self-efficacy^31^. Despite a correlation of *r* = .59, factor analysis indicates their distinctiveness; hope is positively related to optimism, whereas self-efficacy is not ^33^. Additionally, hope develops from childhood, and its level can be increased through training and psychological interventions^17^, ^34^, ^35^.

### Metacognition

Metacognition, which refers to an individual’s knowledge about their own cognitive processes and the ability to regulate these processes, is a key area of research in psychology^36^–^36^. Metacognition encompasses the monitoring and control of thinking^41^, and its research finds applications in neurocognition^42^, working memory^43^, assessment^44^, decision-making^45^, cognitive development in children^46^–^36^, problem-solving^49^–^36^, and critical thinking processes^52^.

Various measures of metacognition are presented in the literature, but not all accurately reflect an individual’s metacognition^53^. Schwartz and Metcalfe emphasise the importance of awareness of the limitations of prediction accuracy, which resembles the concept of epistemic humility. The authors also focus on metamemory and assessing the feeling of knowing^54^. Intelligence does not always equate to wisdom, and some researchers use different indicators to assess metacognitive monitoring accuracy^55^. Although we are not focusing on metacognitive monitoring here, we note its importance in the broader metacognitive landscape.

We define metacognition as an individual’s self-awareness regarding biases (metacognitive self – MCS, as part of metacognition^56^). According to Efklides^57^, the monitoring function has two aspects: metacognitive knowledge and metacognitive experiences. Metacognitive knowledge is declarative knowledge, encompassing beliefs about people (including oneself), goals, tasks, and strategies. Metacognitive experiences are feelings and evaluations related to task processing, such as the sense of difficulty or effort assessment^57^. Thus, metacognition includes metacognitive knowledge (here: self-awareness of biases) and metacognitive experiences, which are the first step towards forming metacognitive thoughts. These experiences result from the dynamics of information processing at the object level^4^. Metacognitive thoughts depend on the availability of a schema and the perception of the material as easy or difficult. The metacognitive experience of ease or difficulty is often referred to as conceptual fluency^58^. We assume that for some individuals, the experience of a biased self may be easy to assimilate, while for others, it may be difficult. However, according to Efklides and colleagues^59^, metacognitive knowledge about effort is associated with control strategies and behaviours. We suspect that through a certain form of personal intellectual humility towards oneself, the metacognitive experience of a biased self may be part of metacognitive knowledge about oneself and lead to strong self-awareness of biases.

Many educational studies underscore the vital role of metacognition in learning. For instance, Džinović et al.^60^ demonstrated that metacognition, combined with self-efficacy and motivation, predicts academic achievement, with self-efficacy mediating the effect of metacognition on performance. Self-efficacy also influences students’ metacognition in virtual learning environments, encouraging help-seeking, strategy application, and time management^61^. Training in metacognitive strategies has been found to reduce academic procrastination and boost self-efficacy^62^, while metacognition and self-efficacy are critical components in self-regulated learning, especially in mathematics^63^, ^64^. Chen et al.^65^ proposed a “strategic mindset” distinct from self-efficacy, self-control, grit, and growth mindsets, explaining unique variances in metacognitive strategy use. Berry^66^ cited Bandura’s view on self-efficacy judgments as vital determinants (or results) of human cognition, including metacognition.

The concept of the interrelationships between metacognition, self-efficacy, and hope seems to be embedded in studies on academic goal achievement, where MCS plays a pivotal role. Verbal cognitive ability significantly affects early metacognitive knowledge, which, in turn, influences later gains in reading comprehension^67^. Edossa explored how metacognitive strategies support reading comprehension and the developmental interplay between these constructs, emphasising metacognitive knowledge’s role within metacognitive monitoring and experiences^68^, ^69^.

It is assumed that metacognitive experience, related to metacognitive feelings and the level of internal epistemic motivation, shapes an individual’s awareness of biases^70^. Our study focuses on a particular type of metacognition – self-awareness of biases, which are principles of thinking, evaluating, decision-making, attribution, and behaviour, mainly anchored in heuristics^71^, ^72^. Examples include positivity bias, confirmation bias (seeking information that aligns with pre-existing beliefs^57^, ^74^), and optimism biases, such as overestimating future success or underestimating task completion time^75^–^77^. Accurate self-perception of biases requires acknowledgement of these biases in one’s behaviour. MCS, rooted in intrinsic motivation, reflects a deliberate, reflective self-assessment^78^, where high-MCS individuals tend to seek self-diagnostic information, fostering self-knowledge and psychological improvement^79^–^81^. Moreover, experimental studies indicate^56^ that individuals with high MCS have a greater need for achievement and are more accepting of autonomy and achievement.

MCS positively correlates with similar constructs, such as self-regulatory metacognition^82^ and other metacognitive constructs that enhance positive emotions^83^. Further studies have shown negative correlations between MCS and maladaptive metacognition, such as positive beliefs about worrying, a strong tendency to ruminate, and other thoughts related to depression and various mental disorders^84^. Studies have shown that MCS plays an adaptive role and is negatively correlated with rumination and mental disorders and positively with emotional stability and agreeableness^85^. Thus, a strong MCS reflects an individual’s capacity to acknowledge psychological patterns, biases, and illusions, often correlated with positive dispositions and emotions.

Our study investigates the relationships between MCS, self-efficacy, and hope, positing MCS as a potential predictor of both constructs. Observing students’ goals and motivations at a given time provides insight, but examining developmental trajectories, including beliefs and expectations such as hope and self-efficacy, yields a more holistic perspective. A longitudinal study thus better captures the dynamic interplay among these variables, offering a developmental model more valuable than a single-time assessment.

## Method

### Participants

The participants were recruited randomly from undergraduate students at the Department of Humanities and the Department of Social Sciences of the University of Gdansk. In this study, sample size determination involved considering both between-group differences and interaction effects in repeated measures analysis of variance. For between-group differences, a priori calculation used a medium effect size (f) of 0.20, alpha (α) of 0.05, power of 0.95, two groups, five measurements, and a 0.5 correlation among measures, yielding a total sample size of 198 with an actual power of 0.951. Simultaneously, a similar analysis for interaction effects in repeated measures ANOVA with within-between interaction used a small effect size (f) of 0.10, α of 0.05, power of 0.95, two groups, five measurements, 0.5 correlation among measures, and a nonsphericity correction (ε) of 0.8, resulting in a total sample size of 220 with an actual power of 0.95. A larger sample size was adopted to ensure a robust and adequately powered study, reflecting meticulous consideration of both between-group differences and interaction effects. We provided sufficient power to analyse even weaker effects than originally assumed with samples from all available students.

Assessments were carried out every six months over the three-year study period, with five assessments overall. Given that some students participated in foreign exchange programs, took leaves of absence, or experienced academic setbacks leading to their exclusion from the university, additional participants were recruited during subsequent phases of the study. The exclusion criteria included observations over 26 years of age, representing 2.6% of the original sample. This exclusion aimed to mitigate potential confounding factors introduced by older students, ensuring a more accurate representation of the targeted age range and minimising the impact of age-related influences on the recorded changes. As for inclusion criteria, participation was contingent solely upon the presence of students on the university campus on the day of data collection. This selective criterion ensured that participants actively engaged with the study, contributing to a representative and meaningful dataset for the research investigation.

The first assessment was completed by 416 students (380:38 – female: male ratio) during the summer semester of the first year, March-April 2014. The second assessment (winter semester of the second year, November 2015) was completed by 395 students (358:37) – this sample consisted of new participants. The third assessment was completed by 352 (321:31) participants (in the summer semester of the second year, April 2015). 345 (313:34) students participated in the fourth assessment (the winter semester of the third year, November-December 2016, and 363 (331:32) students participated in the fifth assessment (the summer semester of the third year, April-May 2016). Gender imbalance comes from recruitment occurring in the humanities and social sciences departments, where a huge disproportion between the number of studying women and men occurs. Participants’ mean age at the first wave was 20.1 years (*Md* = 20, *SD* = 2.66), and the age range across all time points was 19–25 (*M* = 20.9, *Md* = 21, *SD* = 1.15).

## Materials

We used three questionnaires to assess the level of selected properties. The measurement models for GSE, hope, and MCS were defined based on their theoretical frameworks. For GSE, a single-factor model with ten items was used, where each item was loaded onto the latent construct of general self-efficacy. Similarly, hope was assessed through a two-factor model (agency and pathways), with items loading on respective subdimensions. For MCS, a single-factor model was applied as per established guidelines.

### Metacognitive self questionnaire

The MCSQ-21^85^ contains 21 items describing situations reflecting common biases (e.g., accessibility heuristic: “A view of a murder victim’s body influences my attitude towards the perpetrator more than factual information that a fascist killed thousands of people during the Second World War”^71^, ^72^; positivity bias: “I tend to judge other people positively rather than negatively”^86^). Items are rated on a six-point Likert scale (1 = “totally disagree” to 6 = “totally agree”), reflecting the extent to which each behaviour applies to them. Each item reflects one common bias. The bias is expressed in an episodic way. Participant consciously states whether the given episodic behaviour (bias) relates to their own repertoire of behaviours, where one means: “definitely not” up to six: “definitely yes”.

The model-based omega reliability coefficient^87^, ^88^ for the general MCSQ-21 factor and the internal consistency (Cronbach’s alpha) of the MCSQ-21 in our sample throughout the study was satisfactory; values at the five waves were presented in Table 1. Construct validity estimated from RMSEA for the bi-factor model was also at a satisfactory level and was presented in Table 1.

**Table 1:** Psychometric properties of MSCQ-21 for five-time points.

	Alpha	Omega_t_	Variance explained	RMSEA [90% CI]
Wave 1	0.69	0.74	23.7%	0.042 [0.035, 0.049]
Wave 2	0.73	0.78	26.7%	0.055 [0.049, 0.061]
Wave 3	0.77	0.81	30.8%	0.056 [0.051, 0.062]
Wave 4	0.76	0.82	31.0%	0.055 [0.049, 0.061]
Wave 5	0.78	0.83	33.4%	0.057 [0.051, 0.063]

Alpha (Cronbach’s), Omega_t_ (McDonald’s) – reliability indices; Variance explained based on factor analysis with a minimum residual solution; RMSEA – robust root mean square error of approximation with Satora-Bentler correction.

### Generalised self-efficacy scale

The Polish version of Schwarzer and Jerusalem’s^89^ GSES was used to measure general self-efficacy^90^. The questionnaire contains ten items (e.g., “Thanks to my resourcefulness, I can handle unforeseen situations,” “It is easy for me to stick to my aims and accomplish my goals”). Possible responses are 1 (“not at all true”), 2 (“hardly true”), 3 (“moderately true”), and 4 (“exactly true”). The psychometric properties of the GSES scale across all five studies are at least satisfactory (Table 2).

**Table 2:** Psychometric properties of GSES for five-time points.

	Alpha	Omega_t_	Variance explained	RMSEA [90% CI]
Wave 1	0.85	0.85	100%	0.059 [0.042, 0.077]
Wave 2	0.85	0.85	100%	0.075 [0.059, 0.091]
Wave 3	0.88	0.88	100%	0.055 [0.038, 0.072]
Wave 4	0.89	0.89	100%	0.064 [0.047, 0.081]
Wave 5	0.91	0.91	100%	0.076 [0.060, 0.092]

Alpha (Cronbach’s), Omega_t_ (McDonald’s) – reliability indices; Variance explained based on factor analysis with a minimum residual solution; RMSEA – robust root mean square error of approximation with Satora-Bentler correction, CFA with free parameters on residuals correlation between item 4 & item 6.

### Adult dispositional hope scale

The adult dispositional Hope Scale^28^ contains four items measuring agency (e.g., “I energetically pursue my goals”), four measuring pathway thinking (e.g., “Even when others get discouraged, I know I can find a way to solve the problem”), and four items measuring distracters. Each item is rated on an 8-point Likert scale ranging from “1 = definitely false” to “8 = definitely true “. The total Hope Scale score evaluates how they perceive themselves when pursuing their goals in different situational contexts. This study used the Polish version^57^ of the scale. The psychometric properties of the Hope Scale across all five studies are at least satisfactory (see Table 3).

**Table 3:** Psychometric properties of Hope Scale for five-time points.

	Alpha	Omega_t_	Variance explained	RMSEA [90% CI]
Wave 1	0.75	0.80	34.7%	0.073 [0.061, 0.086]
Wave 2	0.80	0.84	28.8%	0.053 [0.040, 0.066]
Wave 3	0.83	0.86	30.5%	0.068 [0.056, 0.080]
Wave 4	0.82	0.85	31.9%	0.069 [0.058, 0.081]
Wave 5	0.81	0.86	26.4%	0.053 [0.041, 0.065]

Alpha (Cronbach’s), Omega_t_ (McDonald’s) – reliability indices; Variance explained based on factor analysis with a minimum residual solution; RMSEA – robust root mean square error of approximation with Satora-Bentler correction. Residual covariance allowed on item 3 & item 11, item 7 & item 11 and item 3 & item 7.

### Procedure

Students were informed of the study’s overall goal and the use and confidentiality of their data and signed a consent form. All individuals agreed to disclose their data to researchers. They also agreed to attend study appointments in person. A trained research assistant or an investigator administered the session. Study appointments were scheduled close to the end of each semester but before the final exam period and took place individually or in groups of up to 30 students. Each time, participants were asked to follow the instructions of the test battery. After providing their demographic data, participants were given three questionnaires – the Metacognitive Self Questionnaire^85^ , Generalized Self-Efficacy Scale (GSES^90^), and Adult Dispositional Hope Scale^91^ – in random order. We used the same instruments in each of the five studies. The effect of the randomisation was non-significant: F < 1. We conducted a paper-pencil study to ensure that participants complied with its procedures. Participants were fully debriefed at their last appointment (if no further participation was possible or they completed wave V). No incentives for participation in the study were offered. The entire study procedure was controlled by the University’s Confidentiality Office and was approved by the Ethical Committee no. 17a/2013, according to Helsinki Demand.

The longitudinal study needs each person to complete the same questionnaires step by step, and all measures are treated as repeated measures. Our study fulfils the fundaments of a longitudinal study. Notably, the 257 students who participated in all five measurements did not exhibit significant differences in scores from those who additionally participated in the intermittent assessments, ensuring the consistency and robustness of our longitudinal design.

## Statistical analyses

We began by analysing the association between MCS and both GSES and Hope scores using partial correlation coefficients, controlling for time as a covariate, with confidence intervals computed via bootstrapping. A longitudinal confirmatory factor model (CFA) with Satorra-Bentler scaled chi-square and robust standard error corrections was then fitted to address any distortions from univariate and multivariate non-normality, with model fit assessed via RMSEA (< 0.06), SRMR (< 0.08), and CFI (> 0.95), as per the recommendations of Hu and Bentler^92^. We subsequently modelled the learning curves as linear, with a constant effect of MCS across time points, incorporating random effects for slope and intercept. The effect of MCS on model fit was assessed by comparing nested models using a -2 log-likelihood difference. Following these steps, the general learning curve results guided us to perform a causal mediation analysis to explore the potential indirect effect of MCS on GSE and a regression analysis to examine the moderating effect of MCS on Hope scores. All analyses were conducted using R version 4.0.4 with the lavaan package (v0.6.8), lme4 package (v1.1-26), and mediation package (v4.5.0).

We analysed the influence of MCS on the change in GSES and Hope scores across the five-time points. In the first step of the analysis, we tested the existence of associations between MCS, GSES, and Hope with partial correlation coefficients with time as a covariant and confidence interval computed using the bootstrap method. In the second step, we fitted a longitudinal confirmatory factor model with fixed covariate (CFA) with Satorra-Bentler scaled *chi*^*2*^ and robust SEs corrections to provide more accurate parameter estimations for distortion from univariate and multivariate normality. Model fit was evaluated with the root mean squared error of approximation (RMSEA < 0.06), standardised root mean square (SRMR < 0.08) and comparative fit index (CFI > 0.95), as recommended by Hu and Bentler ^92^. Next, the general learning curves were modelled as linear with a constant effect of the MCS at each time point and random effects for the slope and intercept. The effect of the MCS on the model fit was estimated via a comparison of nested models. The change in model fit was estimated according to a -2 log-likelihood. The analyses exploring the mediating and moderating roles of MCS were carried out in an exploratory manner to examine the potential indirect effect of MCS on GSES and the effect of MCS on Hope scores. These analyses were carried out using R version 4.0.4 using the lavaan package (version 0.6.8), the lme4 package (version 1.1–26) and the mediation package (version 4.5.0).

## Results

The grand mean total scores of the MCSQ-21, GSES, and Hope Scale scores were as follows: MCSQ-21 (*M*_*grand*_ = 4.33, *SD* = 0.45, *range* 1.57–5.67), GSES (*M*_*grand*_ = 3.06, *SD* = 0.45, *range* 1.40–4.70), and Hope Scale scores (*M*_*grand*_ = 4.66, *SD* = 0.70, *range* 2.08–6.67).

Weak correlations were found between the MCSQ-21 and GSES (*r* = .18, *CI*_*.95*_ = [0.08, 0.27]) and Hope Scale scores (*r* = .26, *CI*_*.95*_ = [0.18, 0.35]). However, cross-sectional data cannot be used to build a theoretical model of dynamic relations. Therefore, we used growth curve analysis to examine the changes in participants’ GSES and Hope Scale scores and to what extent MCSQ-21 scores predicted changes in the other two variables.

**Fig. 1:**
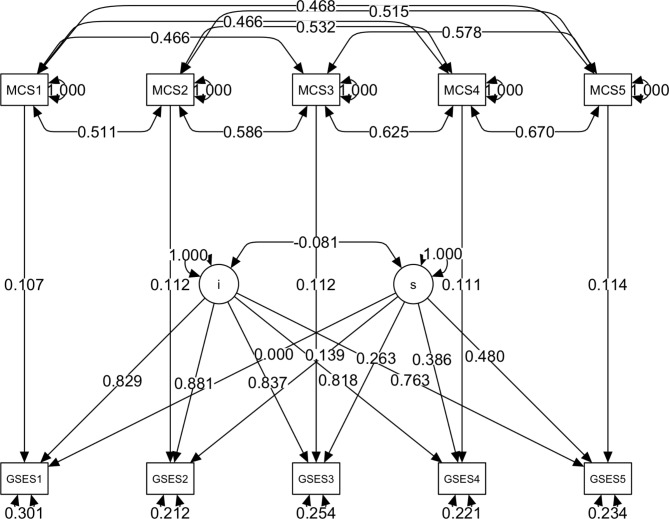
Longitudinal growth curve model with fixed covariate for self-efficacy (GSES); MCS stands for metacognition self; numbers in variable names are for time points; i – intercept, s – slope.

**Fig. 2:**
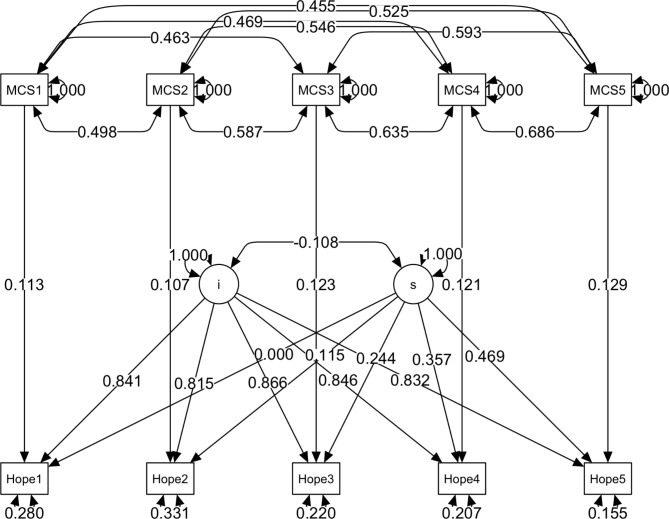
Longitudinal growth curve model with fixed covariate for Hope; MCS stands for metacognition self; numbers in variable names are for time points; i – intercept, s – slope.

As depicted in Figs. 1 and 2 and a latent growth curve model was estimated to investigate changes in self-efficacy and hope over 2.5 years (five waves of data separated by half years each). The model set each intercept factor loading equal to 1 and the slope factor loadings equal to 0, 1, 2, 3, and 4. For self-efficacy, the model fits the data well according to the relative fit indices, with *chi*^*2*^(34) = 42.535, *CFI* = 0.991, *SRMR* = 0.064, *RMSEA* = 0.032, *CI*_*.90*_ = [0, 0.060]. The mean of the intercept factor for self-efficacy was 2.986, nearly identical to the observed mean for the first wave (3.003). The mean of the slope factor was 0.024, *p* < .001, indicating a significant increase of 0.024 points on the GSES score every half year.

**Fig. 3:**
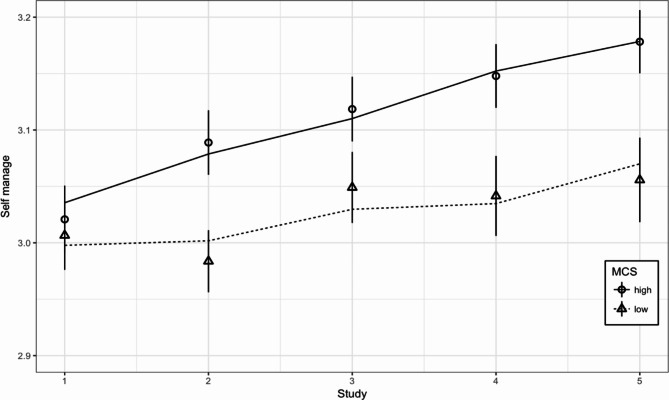
Changes in average Generalized Self-Efficacy Scale score among people with Metacognitive Self Questionnaire (MCS) scores below (low) and above (high) the median.

The MCSQ-21 score was a significant factor influencing the model constant (*chi*^*2*^(1) = 25.59, *p* < .001), indicating that MCS significantly influenced the change in self-efficacy over the study period. However, the effect was weak, with a 0.024 (SE = 0.01) increase in GSES scores per point of difference in MCS every six months. Moreover, there was a slightly faster increase in GSES scores among those with higher MCS levels than those with lower MCS levels (*chi*^*2*^(1) = 3.85, *p* = .0498). The data and model fit for the GSES are shown in Fig. 3.

After seeing that MCS had an effect on intercepts of GSES, a causal mediation analysis was used to estimate the extent to which the MCS accounted for the development of GSES. For this analysis, confidence intervals were estimated using the bootstrap method. The total effect of time (*beta* = 0.028, *CI*_*.95*_ = [0.014, 0.040]) was slightly mediated by MCS score (*beta* = 0.004, *CI*_*.95*_ = [0.002, 0.010]). The indirect effect accounted for only 20.4% (*CI*_*.95*_ = [8.3, 53.0]) of the total effect.

For the latent curve growth model, the mean of the intercept factor for the Hope scale was 4.917 and significantly increased by 0.027 every half year. The model fit the data well according to the relative fit indices, with *chi*^*2*^(34) = 56.795, *CFI* = 0.976, *SRMR* = 0.086, *RMSEA* = 0.055, *CI.90* = [0.028, 0.080]. The relatively large variance for the slope factor in the Hope score (0.012, for GSES, was only 0.003) and the difference with the average result of the first wave (mean was 4.995) led us to look for groups of trajectories of change and to investigate the moderating effect of MCS on Hope.

**Fig. 4:**
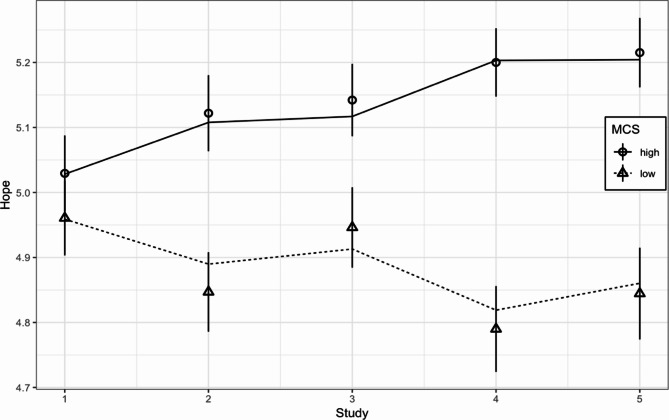
Change in average Hope Scale scores over time among those with Metacognitive Self Questionnaire (MCS) scores below (low) and above (high) the median.

The interaction effect of MCS on Hope was significant (*beta* = 0.099, *SE* = 0.030, *p* < .001), indicating that the level of hope increased faster among individuals with above-median MCS scores, while those with MCS scores lower than the median showed an irregular decrease in hope (Fig. 4).

## Discussion

The findings indicate that MCS serves as a resource that supports the development of both self-efficacy and hope, suggesting potential pathways for promoting this growth. Interestingly, MCS has a linear influence on GSE: the higher the score on the MCSQ-21, the faster the development of self-efficacy. However, for hope, the relationship is more complex. MCS can act as a moderator, enhancing hope only when an individual reaches a certain MCS level; below this threshold (the median), participants’ hope decreases over time. Therefore, increasing MCS may foster a stronger sense of self-efficacy and hope.

It is important to emphasise that MCS is a component of metacognition. Szczepanik and colleagues^93^ measured metacognitive strength using MCS alongside hedonic abilities (with a scale for anticipatory and consummatory anhedonia) and investigated how individuals with high and low MCS levels assess potential pleasure from engaging in various activities using an fMRI task. This task involved evaluating the potential pleasure of presented activities (via images or words), and BOLD signals were recorded during each positive decision. Research into the neural correlates of hedonic decision-making and MCS suggests that the signal in several cortical areas associated with higher-order processes was positively correlated with higher MCS levels. In particular, activation was observed in the temporoparietal junction, a region associated with social cognition, empathy, perspective-taking, and prosocial behaviours^94^–^97^. Other areas where BOLD activity during hedonic evaluations was linked to MCS scores include regions involved in motor processes, imitation, embodied cognition, and reward evaluation^98^–^100^. Thus, during hedonic decisions, the “metacognitive self” is associated with recruiting neural resources necessary for task performance. This study complements Foxall’s findings^92^ regarding a decision-making system based on the prefrontal cortex, which serves a metacognitive role in behaviour control by integrating reflective and rational processes. In line with recent research on modelling cognitive control, contrasting Type 1 processing (rapid, autonomous) with Type 2 processing (slower, computationally demanding), the authors’ model presents a space where impulsive and executive aspects of decisions may be considered, leading to more appropriate choices. Gold^102^ posits that single neurons and neural networks will contribute to a fuller understanding of cognition. Szczepanik et al.’s study^93^ confirms that the “metacognitive self” is rooted in higher-order processes and linked with positive emotions.

Having established that MCS is an aspect of metacognition, we return to discuss our findings. A potential explanation for the associations between MCS and hope is that MCS correlates with positive metacognitive emotions^83^. Hope reflects belief in a better future, and hopeful individuals remain optimistic. Research on hope^102^, ^103^ describes how hope develops through learning connections between actions and outcomes and forming beliefs about these relationships. Successes in problem-solving can lead to beliefs in self-efficacy, while failures may gradually reduce hope levels^104^. In previous studies on hope-enhancing interventions, Lopez suggested focusing on cognitive skill training, such as mental scenario rehearsals or practical guidance^92^.

Our findings suggest that real-life challenges may foster hope development only in individuals with stronger metacognitive skills. Conversely, the decline in hope observed among participants with low MCS may result from repeated failures experienced by those students who held overly optimistic yet unrealistic expectations about their goals and future. Individuals with low MCS may struggle with accurately assessing difficulty and effort^57^, especially in the new context of beginning university studies. In some cases, high levels of hope combined with low MCS may lead to setting overly ambitious goals and difficulties in monitoring progress, limiting self-regulation effectiveness^106^ and exposing them to more frequent setbacks. Hope develops throughout life and is experience-dependent, so when experiences outweigh successes, hope levels may decrease. Therefore, it is worth considering metacognition as a resource that protects against misjudging situations and one’s abilities.

The difference between GSE and hope, evident in the results, is intriguing. Both variables are associated with MCS and increase over time, but only in the case of hope does MCS alter this trajectory. At the same time, research^33^, ^92^ suggests that “agency” is more strongly associated with self-efficacy than “pathway thinking”. The differing effects of MCS on hope and self-efficacy may stem from pathway thinking – a core element of hope but not self-efficacy.

It should be noted that at the start of the study, hope did not significantly differ between low- and high-MCS groups. However, this difference gradually grew, possibly due to the specific nature of metacognitive experiences at this life stage. Early adulthood is a time for identity formation and making significant decisions, such as selecting learning strategies.

The impact of high MCS on hope and self-efficacy levels can also be explained by the frequency of errors made by participants. Individuals with higher MCS are more aware of their errors than those with lower MCS levels^92^, ^92^, and they also more frequently receive feedback from their environment^92^, which facilitates better learning. In other words, more learning opportunities lead to greater increases in hope and self-efficacy than in individuals with low MCS. Self-regulated learning develops through MCS’s influence (awareness of one’s errors and illusions) on GSE (motivation) and hope (affect, abilities, beliefs).

**Limitations and practical implications**. The results are based on self-reported data – we know only the declared levels of hope and self-efficacy, so behavioural indicators of these beliefs would be useful. Additionally, participants who dropped out of the study for various reasons may have differed from those who remained, which is a common issue in longitudinal studies. However, in our case, analysis of the results of those who participated in all measurements (*N* = 257) with the remaining participants did not show significant differences.

We are aware that the results may be weaker than we believe because we did not control for all possible factors that could have influenced the variables studied. However, our findings suggest that intervention programmes could significantly shape self-knowledge by raising awareness of biases (e.g., implicit bias training). The results indicate that experiencing situations where biases may affect perception and decisions leads to greater self-insight, which ultimately supports the development of self-efficacy and hope for success. Therefore, enhancing metacognitive competence may provide substantial support in dealing with challenges in educational and occupational contexts.

**Author contributions**.

Conceptualisation, HB, PK; methodology, PK, HB; software, PK; validation, AF, MZ, HB and PK; formal analysis, PK; investigation, AF, HB; resources, HB, MZ; data curation, AF, PK; writing—original draft preparation, HB, PK, AF and MZ; writing—review & editing, HB, PK, AF and MZ; visualisation, PK; supervision, HB, PK; project administration, AF; funding acquisition, HB.

Funding.

Grant from National Centre of Science (2013/11/B/HS6/01463) for Hanna Brycz.

## Data Availability

The data analysed during the current study are available from the corresponding author upon reasonable request.
